# Study of insecticidal and fungicidal potential of endophytic bacteria of wheat, soybean and rapeseed by bioinformatic analysis methods

**DOI:** 10.18699/vjgb-25-137

**Published:** 2025-12

**Authors:** T.N. Lakhova, A.I. Klimenko, G.V. Vasiliev, E.Yu. Gyrnets, A.M. Asaturova, S.A. Lashin

**Affiliations:** Institute of Cytology and Genetics of the Siberian Branch of the Russian Academy of Sciences, Novosibirsk, Russia Novosibirsk State University, Novosibirsk, Russi; Institute of Cytology and Genetics of the Siberian Branch of the Russian Academy of Sciences, Novosibirsk, Russia; Institute of Cytology and Genetics of the Siberian Branch of the Russian Academy of Sciences, Novosibirsk, Russia; Federal State Budgetary Scientific Institution “Federal Research Center of Biological Plant Protection”, Krasnodar, Russia; Federal State Budgetary Scientific Institution “Federal Research Center of Biological Plant Protection”, Krasnodar, Russia; Institute of Cytology and Genetics of the Siberian Branch of the Russian Academy of Sciences, Novosibirsk, Russia Novosibirsk State University, Novosibirsk, Russia

**Keywords:** bioinformatics, comparative genomics, endophytes, biocontrol, биоинформатика, сравнительная геномика, эндофиты, биоконтроль

## Abstract

Endophytic bacteria play a key role in agricultural ecosystems, as they can affect the availability of various compounds, crop yield and growth, and provide resistance to diseases and pests. Therefore, the study of endophytes of agriculturally important crop plants is a promising task in the field of biological plant protection. Understanding the mechanisms of interaction between endophytic bacteria and plants will allow the use of these microorganisms as bioagents in the future and thus reduce dependence on chemical pesticides. In this paper, samples obtained from the leaves and/or roots of wheat, rapeseed and soybean are considered. Whole-genome sequencing of the isolates was performed. Using an analytical pipeline, the genomes of 15 strains of endophyte bacteria of cultivated plants were assembled and characterized. Their insecticidal and fungicidal potential was analyzed. Gene repertoire analysis performed with GenAPI showed a high degree of correspondence between the gene repertoires of strain BZR 585 against Alcaligenes phenolicus, BZR 762 and BZR 278 against Alcaligenes sp., BZR 588 and BZR 201P against Paenochrobactrum pullorum. All strains, with the exception of BZR 162, BZR 588 and BZR 201P, were found to contain genes encoding proteins with fungicidal activity, such as iturins, fengycins and surfactins. All strains also contained genes encoding proteins with insecticidal activity, namely GroEL, Spp1Aa1, Spp1Aa2, Vpb1Ab1, Vpb4Ca1, HldE, mycosubtilin, fengycin and bacillomycin. The obtained genomic data are confirmed by the results of previous experimental studies: high insecticidal activity of a number of strains (BZR 1159, BZR 936, BZR 920, etc.) against Galleria mellonella, Tenebrio molitor and Cydia pomonella, as well as fungicidal properties against Fusarium, Alternaria, Trichothecium, was demonstrated. This shows the practical significance of the identified genetic determinants for the creation of new biocontrol agents.

## Abstract (ru)

Эндофитные бактерии способны влиять на доступность различных соединений, урожайность и рост сельскохозяйственных растений, а также обеспечивать устойчивость к болезням и вредителям. Поэтому исследование эндофитов сельскохозяйственно значимых культурных растений является перспективной задачей в области биологической защиты растений. В данной работе рассмотрены изоляты штаммов бактерий, полученные из листьев и/или корней пшеницы, рапса и сои. Было произведено полногеномное секвенирование изолятов. С помощью аналитического конвейера собраны и охарактеризованы геномы 15 штаммов бактерий- эндофитов культурных растений, проанализирован их инсектицидный и фунгицидный потенциал. Анализ генного репертуара с помощью программы GenAPI показал высокую степень соответствия между генным репертуаром штамма BZR 585 относительно Alcaligenes phenolicus, BZR 762 и BZR 278 относительно Alcaligenes sp., BZR 588 и BZR 201P относительно Paenochrobactrum pullorum. Во всех штаммах, за исключением BZR 162, BZR 588 и BZR 201P, найдены гены, кодирующие белки, обладающие фунгицидной активностью, такие как итурины, фенгицины и сурфактины. Также во всех штаммах найдены гены, кодирующие белки с инсектицидной активностью, а именно: GroEL, Spp1Aa1, Spp1Aa2, Vpb1Ab1, Vpb4Ca1, HldE, фенгицин, микосубтилин и бацилломицин. Полученные геномные данные подтверждены экспериментальными испытаниями: ранее показана высокая инсектицидная активность штаммов BZR 1159, BZR 936, BZR 920 и др. против Galleria mellonella, Tenebrio molitor и Cydia pomonella, а также фунгицидные свойства против Fusarium, Alternaria, Trichothecium. Это демонстрирует практическую значимость выявленных генетических детерминант для создания новых агентов биоконтроля

## Introduction

Endophytic bacteria are components of the plant microbiome that can colonize roots, stems, or leaves, where they obtain stable sources of nutrients and, in turn, increase plant resistance to both biotic and abiotic stresses. These symbionts have growth-stimulating activity due to nitrogen fixation, phosphorus mobilization, and phytohormone synthesis, and also produce a wide range of metabolites, hydrolytic enzymes, and volatile compounds involved in the biological control of pests and diseases of agricultural crops. In addition, endophytes compete for ecological niches and induce systemic resistance in plants, creating a multi-level defense that can be comparable in effectiveness to chemical pesticides, but is safe for humans and the environment (Hamane et al., 2023; Ali et al., 2024). The study of endophytes in agriculturally important crops opens up prospects for the search for biocontrol agents for various pests and pathogens (insects, fungi, and others).

Endophytic bacteria are components of the plant microbiome that can colonize roots, stems, or leaves, where they obtain stable sources of nutrients and, in turn, increase plant resistance to both biotic and abiotic stresses. These symbionts have growth-stimulating activity due to nitrogen fixation, phosphorus mobilization, and phytohormone synthesis, and also produce a wide range of metabolites, hydrolytic enzymes, and volatile compounds involved in the biological control of pests and diseases of agricultural crops. In addition, endophytes compete for ecological niches and induce systemic resistance in plants, creating a multi-level defense that can be comparable in effectiveness to chemical pesticides, but is safe for humans and the environment (Hamane et al., 2023; Ali et al., 2024). The study of endophytes in agriculturally important crops opens up prospects for the search for biocontrol agents for various pests and pathogens (insects, fungi, and others).

For example, in the rhizosphere of plants, B. velezensis creates a favorable nutritional and physicochemical environment for root microbiota by forming a biofilm, which promotes plant growth and protection against phytopathogens, both through the secretion of antimicrobial compounds and through the formation of phytoimmune potential in plants. The study (Rabbee et al., 2019) summarized information on strain-specific gene clusters of B. velezensis associated with the biosynthesis of secondary metabolites, which play an important role in both suppressing pathogens and stimulating plant growth. For B. velezensis BRI3 strains and related lines, comparative analysis confirmed the preservation of the “core” gene clusters of lipopeptide synthetases (iturin, fengycin, and surfactin), with the simultaneous appearance of unique type III polyketide synthases, which allows these strains to exhibit broad fungicidal potential in vitro (Liu et al., 2024).Pangenomic studies of Burkholderia bacteria, including a comparison of 18 endophytic and pathogenic strains, revealed the loss of classical virulence determinants and enrichment of antimicrobial compound synthesis gene clusters in endophytic symbionts. These changes are a sign of adaptation to an intracellular lifestyle and serve as an indirect marker of biological control potential (Liu et al., 2024).

For the representative of Pseudomonadota, Ochrobactrum quorumnocens A44 is capable of disrupting quorum sensing (QS) in Gram-negative bacteria by inactivating N-acylhomoserine lactones (AHLs) and protecting plant tissues from soft rot pathogens, the virulence of which is regulated by QS. For this strain, isolated from the potato rhizosphere, and six related type strains of the genus Ochrobactrum, comparative genomics showed that the core genome contains 50–66 % of genes, and the variable part for each genome accounts for 8 to 15 % (Krzyżanowska et al., 2019).

The entomopathogenic strain B. thuringiensis ser. israelensis, a well-known source of δ-endotoxins (cry, cyt), plays a key role (occupies a special place) in research. The complete sequence of the 127-kb pBtoxis plasmid showed that the cluster of genes encoding toxins remains stable, while regulatory and mobile elements are actively reorganized, ensuring horizontal transfer of insecticidal protein genes and expanding the adaptive potential of the strain (Berry et al., 2002; Bolotin et al., 2017).

At the same time, microorganisms that establish symbiotic relationships with their hosts were found to contain GroEL proteins (an ATP-dependent molecular chaperone that is present in all forms of life and is one of the most conservative proteins in living organisms), which acted as toxins (Horwich et al., 2007; Shi et al., 2012; Kupper et al., 2014). Its homologue, XnGroEL, has been described in the Xenorhabdus nematophila, which retains its folding function but acquires the ability to bind to the insect’s chitin cuticle and suppress the host’s immune responses (Horwich et al., 2007).

Thus, the creation of databases on proteins associated with the insecticidal and fungicidal properties of endophytic bacteria opens up broad opportunities for bioinformatic analysis and in silico screening of bacteria with protective properties that are significant for agriculture. For example, the BPPRC database (Panneerselvam et al., 2022) summarizes information on some proteins and peptides with insecticidal activity

In this paper, we present assemblies and annotations of the genomes of 15 strains of endophytic bacteria isolated from various organs of wheat, soybean, and rapeseed plants. Annotation was performed relative to close bacterial reference genomes. We also demonstrated the presence of proteins with fungicidal and insecticidal properties in the studied strains

## Materials and methods

Strains of endophytic bacteria. In this research, we used the scientific equipment “Technological line for obtaining microbiological plant protection products of a new generation” (http://ckp-rf.ru/usu/671367). The objects of the study were bacterial strains from the bioresource collection of the Federal State Budgetary Scientific Institution Federal Research Center for Biological Resources “State Collection of Entomophagous Acarids and Microorganisms” (https://fncbzr.ru/brk-i-unu/ unique-installation-1/). The strains under study were isolated from the roots and leaves of wheat, soybeans, and rapeseed. Samples were collected in four districts of the Krasnodar Territory (Krylovsky, Vyselkovsky, Pavlovsky, and Krasnodar). The general characteristics of the microbial strains under study are presented in Supplementary Material 11.

Supplementary Materials are available in the online version of the paper: https://vavilovj-icg.ru/download/pict-2025-29/appx52.zip


Sequencing. Bacterial DNA was isolated from individual colonies grown on agarized medium in Petri dishes using the D-Cells kit (Biolabmix, Russia) according to the method for Gram-negative bacteria. The colony was transferred to a 1.5 ml tube and resuspended in 150 μl of PBS buffer. After adding 20 μl of proteinase K and 150 μl of lysis buffer, the cells were incubated for 10 min at 56 °C. After adding 500 μl of LB buffer, the lysate was applied to the column and centrifuged for 30 seconds at 12,000g. The column was sequentially washed with 500 μl of WB1 and WB2 buffers, followed by centrifugation. DNA was eluted from the column using 60 μl of EB buffer. Ultrasonic DNA fragmentation was performed on a Covaris M220 sonicator (Shelton, USA) in a volume of 50 μl using a protocol optimized for average fragment lengths of 300 bp. DNA concentration was measured using a Qubit4 fluorometer with the DNA HS kit (Thermo Fisher Scientific, USA). Genomic libraries were prepared using the KAPA Hyper Prep kit (Roche, Switzerland) with KAPA double indices according to the manufacturer’s instructions. Fifty nanograms of fragmented DNA were used for the experiment, with nine cycles of final PCR. The quality and molarity of the obtained libraries were calculated after analysis on a BA2100 bioanalyzer (Agilent, USA) and measurement of concentrations on a Qubit4 fluorometer. After normalization, the resulting libraries were pooled to a concentration of 4 nM/μl. Sequencing was performed on a GenolabM device (GeneMind, China) using 2×150 bp paired-end reads with the GenolabM V2.0 kit (FCM 300) according to the manufacturer’s protocol.

Bioinformatic analysis. The genome sequencing results were quality checked using FastQC (https://www.bioinfor matics.babraham.ac.uk/projects/fastqc/), trimming was performed using fastp (Chen et al., 2018; Chen, 2023). Filtering for possible contamination by human reads (Hg38) was performed using BWA MEM (Li, 2013) and synthetic sequences (Univec (https://ftp.ncbi.nlm.nih.gov/pub/UniVec/, accessed April 2025) and blastn).

The taxonomic composition of the sequenced material was analyzed using the MetaPhlan4 tool (Blanco-Míguez et al., 2023). The initial taxonomic identification of the draft primary genome assemblies was performed by tetracorrelation search using the JSpeciesWS web service with the GenomesDB database (Richter et al., 2016). The draft primary assemblies were obtained in the same way as described below, except that Ragout scaffolding (Kolmogorov et al., 2014) and Pilon correction (Walker et al., 2014), were not used for the draft primary assemblies, and the results of the tetra-correlation search during primary taxonomic identification were used as reference genomes. The GFinisher assembler accepts multiple assemblies as input data (Guizelini et al., 2016). The authors of GFinisher note that the first assembly is the main one, and the rest are additional. In our case, depending on the quality control of all assemblies, the main assembly in GFinisher was one of the following: bins, the MinYS assembly (Guyomar et al., 2020), or the Spades assembly (Bankevich et al., 2012; Prjibelski et al., 2020) after mapping to the reference.

**Fig. 1.**
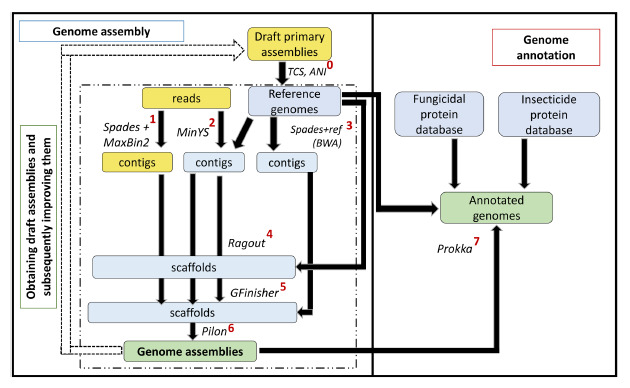
Analytical pipeline for assembling bacterial genomes and annotating them. Yellow blocks indicate data obtained at the preliminary stage of primary genome assembly, blue blocks indicate data obtained during genome assembly refinement, and green blocks indicate target results. Arrows indicate data interdependencies, and labels next to the arrows indicate the tools and methods used for data analysis or conversion. 0 – tetracorrelation analysis (JSpeciesWS and GenomesDB) and average nucleotide identity analysis (fastani) to establish the closest (and best annotated) reference genomes from GenBank; 1 – de novo assembly using Spades followed by binning in MaxBin2 (bin selection filter – completeness > 87 %); 2 – reference-guided assembly in MinYS; 3 – assembly using Spades of reads pre-mapped to the reference using BWA; 4 – scaffolding of previously obtained draft genomes (contigs) in Ragout, using information about the closest reference genome; 5 – genome polishing in GFinisher, combining the results of previously obtained draft assemblies into one common assembly; 6 – final correction in Pilon; 7 – annotation of final assemblies in Prokka, taking into account the most complete known genome annotations of the corresponding species/genus, as well as specially prepared databases of amino acid sequences of fungicidal and insecticidal proteins, the latter including BPPRC.

Next, taxonomic identity was refined in order to select the closest reference genomes for corrected bacterial genome assembly using a hybrid (de novo + reference-guided) cascade approach based on a set of closely related references (Fig. 1). To search for refined reference genomes, average nucleotide identity (ANI) analysis was performed using fastani (Jain et al., 2018) within the genus established during the initial taxonomic screening. Draft primary assemblies were compared by ANI with all publicly available genome assemblies within the same genus deposited in NCBI Genbank, which were downloaded in batch processing using ncbi-genome-download (https://zenodo.org/records/8192486, accessed April 2025).

Three draft assemblies were obtained. The first option was a de novo assembly of Spades. The assembly was binned using MaxBin2 (Wu et al., 2016). The resulting bins were filtered by completeness. If this parameter was <87 %, such bins were not considered further. The second assembly variant was a reference-guided assembly using MinYS. A closely related reference genome and filtered reads were fed into the input. Based on the results of the MinYS assembly, the main reference was selected from the closest reference genomes and used in the third assembly option and in the final assembly with correction. The third assembly option was performed by the Spades assembler based on mapped reads to the selected reference genome.

All draft assembly variants were scaffolded using Ragout, which can utilize information about a number of the closest reference genomes. Configuration files for each Ragout assembly variant were generated using a custom Python 3 script. The resulting draft assemblies at the contig and scaffold level were merged into a single assembly using the GFinisher program. Depending on the intermediate quality control results, the main assembly in GFinisher was either the Ragout assemblies or the bins obtained in the first draft assembly variant. GFinisher can output two assemblies, which were submitted for correction in Pilon. At each stage, the quality of the assemblies was controlled using QUAST (Gurevich et al., 2013). Based on the results of quality control, which was carried out after correction, the final genome assemblies were selected

Genome annotation was performed using the prokka software pipeline (Seemann, 2014), additionally configured to take into account the databases of protein sequences with insecticidal and fungicidal properties prepared within the framework of this study, as well as the annotation of refined reference genomes

For each genome, a separate annotation database was created, consisting of the following parts: annotated protein sequences from the closest reference genome, protein sequences with insecticidal and fungicidal properties collected in this study (described below), and the BPPRC database (https:// www.bpprc-db.org/, accessed June 2025).

To search for insecticidal proteins in the genomes under study, a database of protein sequences was compiled manually. It included the amino acid sequences of Cry and Vip proteins. The sequences were selected from the UniprotDB (Bateman et al., 2021) and NCBI Protein (https://www.ncbi.nlm.nih. gov/protein/, accessed October 2024) databases by genus name and protein name/function. In the complete genomic sequences of bacteria from the GenBank card, the protein product was found and the corresponding protein sequence was downloaded. Then, a search was performed using blastp on the formed database. The results were filtered by a threshold for two parameters: identity > 50 % and e-value < 0.001, where identity corresponds to the percentage of matching amino acids, and e-value corresponds to the statistical significance of the results. The BPPRC database, containing protein sequences of bacterial pesticidal proteins downloaded from the Bacterial Pesticidal Protein Database project, was also added to this variant

Similarly, a database of protein sequences was compiled manually for certain proteins with fungicidal activity: iturins, fengycins, and surfactins. Using blastp, the results of alignment of proteins encoded in the genomes of the analyzed strains to the sequences from the compiled database were obtained. Initial filtering of the results was performed according to two parameters: identity > 50 % and e-value < 0.001

Figure 1 shows a graphical diagram of the corresponding analytical pipeline. Venn diagrams for comparing the gene repertoire of the analyzed strains with that of the reference genomes were constructed using the Draw Venn Diagram web service (http://bioinformatics.psb.ugent.be/webtools/Venn/, accessed May 2025).

Subsequent analysis of the alignment results of the collected genomes based on insecticidal and fungicidal proteins was performed by visualizing information about the proteins found in the studied strains in the form of a heat map based on the iScore value (see Equation 1), which represents the percentage of identity weighted by the proportion of the aligned region to the total length of the reference protein. This representation allows us to take into account not only the percentage of identity of the local alignment found, but also the extent to which this alignment covers the length of the original protein.

The formula for calculating iScore is as follows

**Formula. 1.**
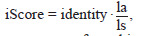
Formula. 1.

where identity is the percentage of matching amino acids, la is the alignment length, and ls is the initial length of the amino acid sequence of the protein from the collected database

Gene repertoire analysis was performed using GenAPI (Gabrielaite, Marvig, 2020).

Multiple alignment of concatenated amino acid sequences was performed using the GTDB-Tk tool (Chaumeil et al., 2022) based on a search for 120 bacterial marker genes. Multiple alignment was used to construct a phylogenetic tree in PhyML (Guindon et al., 2010). In PhyML, the default support level for internal branches is estimated using a Bayesian test. The iTOL web service (Letunic, Bork, 2024) (https://itol.embl.de/, accessed May 2025) was used to visualize the tree.

Program versions and launch parameters are listed in Supplementary Material 2.

## Results

**Taxonomic identification**

A preliminary assessment of the taxonomic composition of sequenced genetic material using Metaphlan revealed heterogeneity in most of the analyzed samples to varying degrees (Table 1). Since not all reads for all samples belonged to a single organism, it was decided to perform binning of the initial draft genome assemblies to isolate the genomic fragments most fully represented in the microorganism sample. Next, taxonomic identification was performed for the bins using tetranucleotide frequency analysis and correlation coefficients (tetra-correlation search, TCS).

**Table 1.**
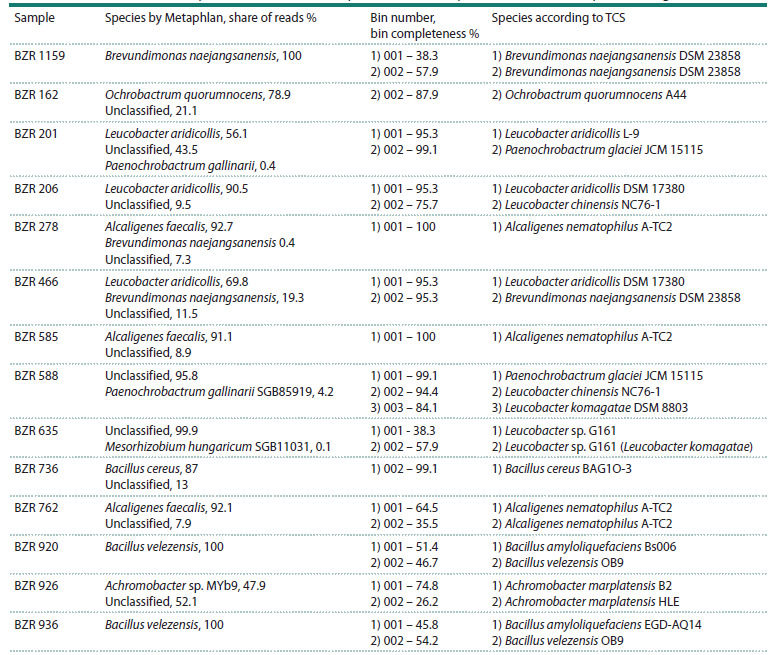
Results of the initial analysis of the taxonomic composition of the samples studied and subsequent binning Notе. The correspondence of the bin numbers in the third and fourth columns has been preserved

**Fig. 2.**
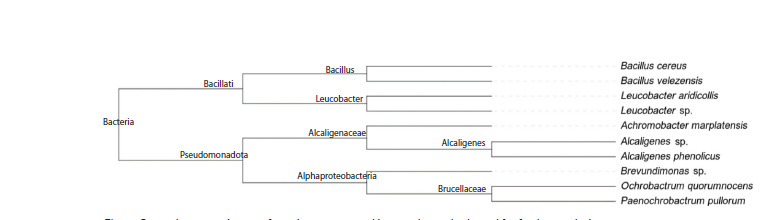
General taxonomic tree of species represented in samples and selected for further analysis

The taxonomic identity of the assembled genomes (see subsection Assembly and annotation) was refined during the analysis of average nucleotide identity by comparison with publicly available reference genomes of the same genus in NCBI Genbank (see Supplementary Material 3). The results of the identification are summarized in Figure 2 and Table 2.

**Table 2.**
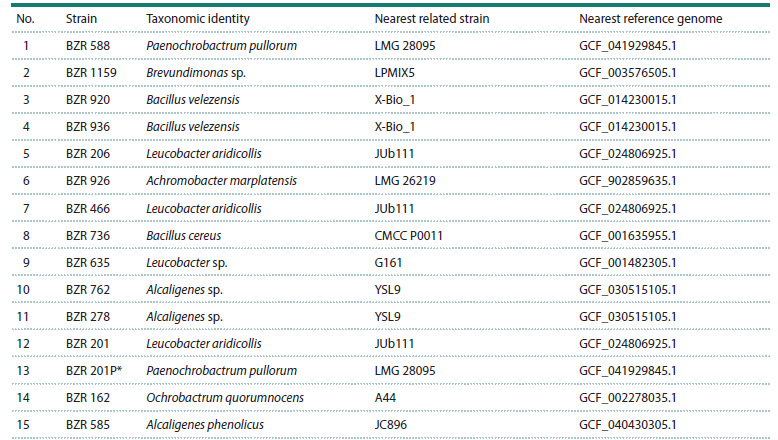
Results of final taxonomic identification with indication of the NCBI identifier of the closest reference genome Note. Since two genomes were isolated from sample BZR 201, the second strain belonging to Paenochrobactrum pullorum from sample BZR 201 is designated BZR 201P (* in the second column

As a result, genomes were assembled and 15 bacterial strains were taxonomically identified. Some of the samples had readings that could be attributed to different bacteria, which may indicate contamination of the samples prior to sequencing or that the culture was mixed

For example, since Paenochrobactrum pullorum is found in samples taken from wheat roots (BZR 588) and winter rapeseed roots (BZR 201P), we conclude that this is not accidental contamination, but a normal representative of the rhizosphere of flowering plants. Thus, in the study (Hussain et al., 2025), this species was used as a part of a community of bacteria that affect phosphorus availability, yield, and wheat growth.

**Assembly and annotation**

As a result of the hybrid (de novo + reference-guided) approach, assemblies of 15 bacterial genomes were obtained. The main characteristics of the final assemblies obtained after QUAST quality control are presented in Table 3. Additionally, for comparison, the table shows such reference characteristics as assembly length (reference genome sequence length), CG composition of the reference genome, and reference-dependent indicators: the proportion of the reference represented in the studied genome (Genome fraction) and the duplication ratio (Duplication ratio).

**Table 3.**
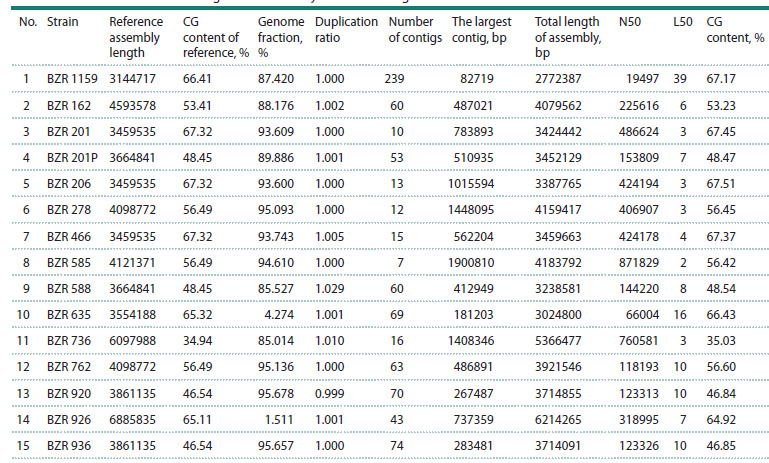
Characteristics of the final genome assembly and reference genome metrics

However, the approach presented was not suitable for samples BZR 635 and BZR 926. Although closely related references were found for these samples, the Genome fraction indicator was low during assembly (4.274 and 1.511 %, respectively), which made it impossible to perform successful scaffolding in Ragout. Therefore, for BZR 635 and BZR 926, the assembly scheme consisted of the following steps. The primary assembly was a de novo Spades assembly without binning (the bins did not pass the set threshold). Rough assemblies were performed using MinYS and Spades with mapping to the reference. The steps with Ragout and Pilon were skipped, since they require the locations of the regions in the assembly relative to the reference. The resulting assemblies were submitted to Gfinisher, the result of which was selected as the final assembly

Genome annotation was performed using the Prokka tool with a user database of annotated amino acid sequences taken from the reference genome, as well as databases of fungicidal and insecticidal proteins. A total of 10 custom databases were used, as the reference genomes coincided for a number of strains. Thus, for strains BZR 201, BZR 206, and BZR 466, the reference is GCF_024806925.1; for BZR 201P and BZR 588 – GCF_041929845.1; for BZR 278 and BZR 762 – GCF_030515105.1; and for BZR 920 and BZR 936 – GCF_014230015.1. Individual user databases were used for the remaining samples

**Fig. 3.**
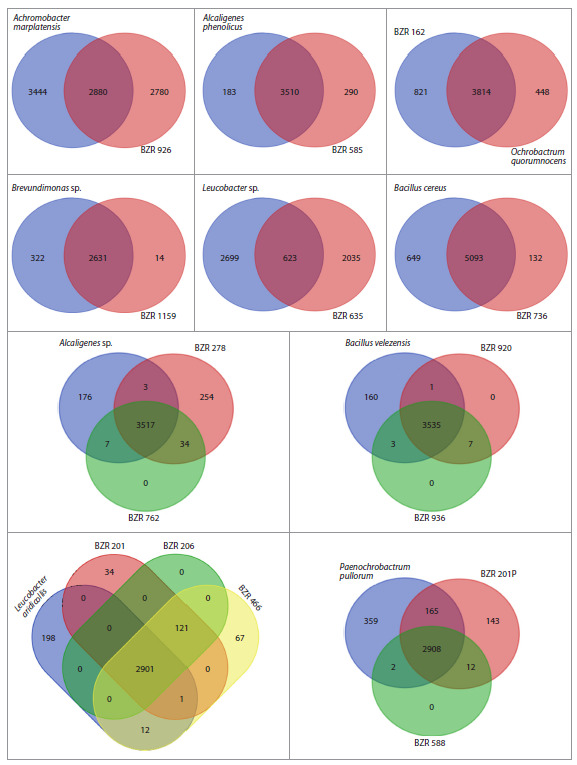
Venn diagrams for gene sets of the studied strains, grouped by species, analyzed together with gene sets from the corresponding reference genomes.

Table 4 shows info on the annotation of bacterial genomes compared to the corresponding reference genome. The “Predicted proteins” column shows the percentage of predicted proteins relative to all genes predicted in the genome, with the rest annotated as “hypothetical protein”. Although the Genome fraction was low for the BZR 635 and BZR 926 genomes, we used the reference data as annotationAnalysis of the gene repertoire using GenAPI revealed differences between the analyzed strains and the reference genomes used (Fig. 3).

**Table 4.**
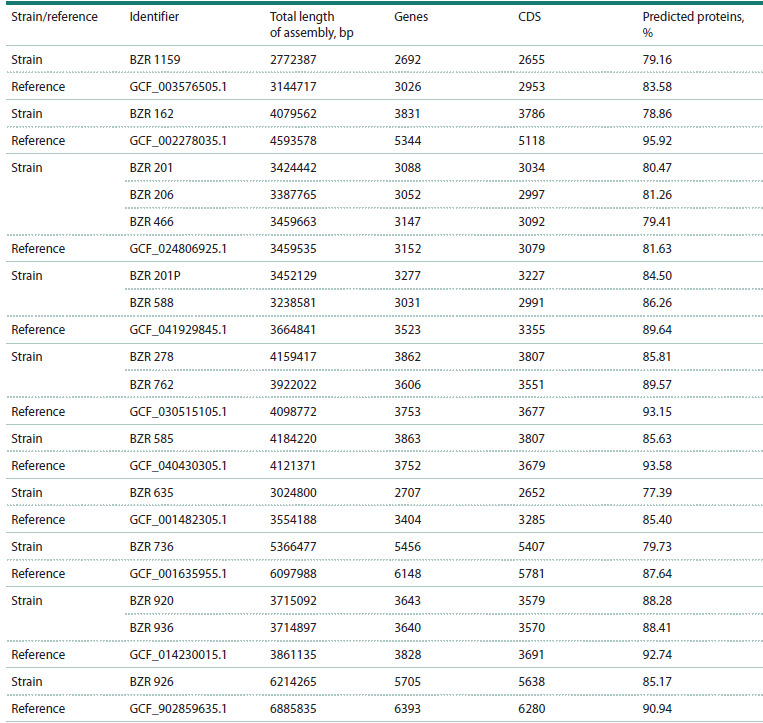
Characteristics of bacterial genome annotations from the analyzed samples compared with the corresponding reference genomes

The genomes of the bacteria were annotated, and the gene repertoire of each genome was analyzed relative to the corresponding reference genomes. Analysis of the intersections of gene sets for different samples showed varying results. For example, strains BZR 920 and BZR 936 showed high similarity to B. velezensis, and strains BZR 206, BZR 466, and BZR 201 showed high similarity to L. aridicollis. However, the fact that 160 genes were found only in the reference genome of B. velezensis but not in the studied strains of this species may be due to both the fragmentation of the BZR 920 and BZR 936 genome assemblies and the accumulated changes in the sequences, based on the homology of which the absence and presence of genes is assessed. It is also possible that these strains do not actually have a number of genes relative to the selected reference genome. These considerations apply to all results presented in Figure 3.

BZR 585 showed a high degree of similarity between the gene repertoire of the studied strains and the corresponding reference genomes in relation to A. phenolicus; BZR 762 and BZR 278 in relation to Alcaligenes sp.; and BZR 588 and BZR 201P in relation to P. pullorum. In addition, BZR 736 showed a good degree of correspondence with B. cereus, BZR 1159 with Brevundimonas sp., and BZR 162 with O. quorumnocens. At the same time, BZR 926 showed only an average level of correspondence with the reference genome of A. marplatensis, and BZR 635, when compared with the closest reference genome, Leucobacter sp., showed only a small number of intersections, which may be due both to different taxonomic identity and to the fragmentation of the genome assembly of this strain.

**Comparison of strains, analysis of their insecticidal and fungicidal potential**

**Fig. 4.**
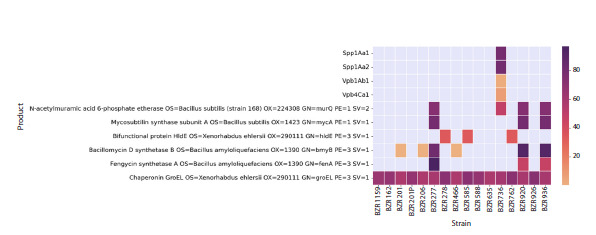
Repertoire of proteins selected during pairwise alignment of translated genomes of the studied strains to the insecticidal protein database, where the color scale reflects the iScore parameter (see Equation 1). That is, the more intense the color, the more completely the sequence from the studied genome was aligned with the amino acid sequence of the protein from the database. Missing proteins are marked in grey. The heat map is based on the values (see Supplementary Material 4).

Below are the results of the analysis of the insecticidal (Fig. 4) and fungicidal (see figure in Supplementary Material 5) potential of the studied strains based on a comparison of their protein repertoires with the corresponding functional activity. Heat maps reflect the results of the search for fungicidal and insecticidal proteins among our strains in the form of iScore, which is the percentage of identity weighted by the proportion of the aligned region of the entire length of the reference protein (see Materials and methods).

A gene encoding the chaperonin GroEl was found in all analyzed strains (Fig. 4). At the same time, the samples belonging to the genus Bacillus (BZR 736, BZR 920, BZR 936) had the highest number of genes encoding insecticidal proteins. While strains BZR 920 and BZR 936, which are representatives of B. velezensis, demonstrate the presence of fengycin, mycosubtilin, and bacillomycin synthetases, BZR 736, belonging to B. cereus, possesses the genes Spp1Aa1 and Spp1Aa2, as well as Vpb1Ab1 and Vpb4Ca1. At the same time, all analyzed strains of the genus Bacillus also possess a gene encoding the enzyme N-acetylmuramic acid 6-phosphate esterase (EC 4.2.1.126). In addition, strains BZR 278, BZR 585, and BZR 762, belonging to the genus Alcaligenes, have fragments homologous to the gene for the bifunctional protein HldE, while strains BZR 201, BZR 206, and BZR 466, representing Leucobacter aridicollis, contain a number of fragments homologous to bacillomycin synthetase genes.

Proteins with fungicidal activity were identified (see Supplementary Material 5; the heat map was constructed based on the values specified in Supplementary Material 6) in samples belonging to the genus Bacillus (BZR 736, BZR 920, BZR 936). It can be seen that the most complete proteins matching the sequences from the database are present in two samples: BZR 920 and BZR 936, belonging to B. velezensis. These samples also lead in terms of the number of proteins found. Since the vast majority of sequences in the database of amino acid sequences of fungicidal proteins belonged to the species B. velezensis and B. amyloliquefaciens, the discovery of proteins in these two samples is a logical consequence. The predominance of data in the collected database of sequences related to B. velezensis and B. amyloliquefaciens was due to the predominance of data on iturins, fengycins, and surfactins of B. velezensis and B. amyloliquefaciens relative to other species of the genus Bacillus in the UniprotDB and NCBI Protein databases. However, genes encoding synthetases, as well as YxjF and YxjC, were also found in a strain belonging to B. cereus (BZR 736), although unlike the other strains analyzed, BZR 736 does not have the ScoA gene, which, along with ScoB, is present in all the strains analyzed. In addition, strains BZR 201, BZR 206, and BZR 466, representing L. aridicollis, contain a number of fragments homologous to the genes of iturin and fengycin synthetases. It is worth mentioning that the genomes of the entire clade of representatives of the Brucellaceae family (strains BZR 162, BZR 588, and BZR 201P) do not contain genes encoding fungicidal proteins represented in our database

**Phylogenetic analysis**

**Fig. 5.**
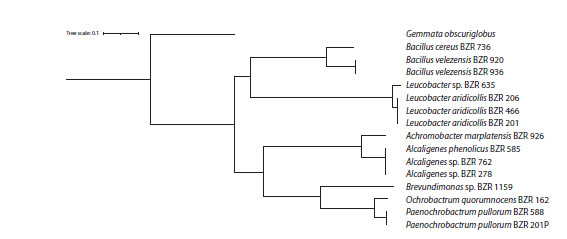
Phylogenetic tree of the studied strains, constructed based on 120 marker genes found in the bacteria. Gemmata obscuriglobus serves as the outgroup.

To construct a phylogenetic tree (Fig. 5), a search for a set of 120 bacterial marker genes was performed using GTDB-Tk in all studied samples, and multiple alignment of protein sequences was performed, which was fed into the tree construction. Proteins from Gemmata obscuriglobus (GCF_003149495.1) were used as an outgroup.

It should be noted that the constructed phylogenetic tree (see Fig. 5) fully reflects the topology of the previously presented taxonomic tree (see Fig. 2), which relatively confirms the correctness of the established taxonomic identity. At the same time, the gene sequences used to construct the tree are identical in strains BZR 762 and BZR 278, as well as in the trio of strains BZR 466, BZR 206, BZR 201, indicating that the former belong to one species of the genus Alcaligenes, and the latter, to L. aridicolis.

## Discussion

The study of endophytic bacteria in agriculturally important crops is a pressing issue, the resolution of which will enable the regulation of pest populations and effective control of plant diseases arising from interaction with pathogens. Therefore, it is particularly important to identify strains that possess fungicidal and insecticidal potential.

To date, the following antagonistic properties of endophytic bacteria are known for the studied genera. Here, we list the genera of bacteria for which taxonomic identification of genomes was performed in this study

Bacillus spp. The genomes of bacteria of the genus B. velezensis contain nine key NRPS/PKS clusters, synthesized lipopeptides (iturin, fengycin, surfactin) and polyketides (deficidin, macrolactin) capable of suppressing phytopathogenic fungi of the genera Fusarium, Bipolaris, Exserohilum, and others, both in vitro and in vivo (Wang S. et al., 2024; Yeo et al., 2024). Surfactin, iturin, and fengycin can act as entomicides and nematicides, as evidenced by the high mortality (up to 100 %) of Aedes aegypti mosquito larvae and Agriotes lineatus click beetles, while pure surfactin causes systemic metabolic disorders in the caterpillars of the Asian cotton bollworm Spodoptera litura (Falqueto et al., 2021; Zhang F. et al., 2024; Knežević et al., 2025).

Alcaligenes spp. A binary protein, AfIP-1A/1B, has been discovered in A. faecalis bacteria that is capable of forming pores in the intestines of western corn rootworm larvae Diabrotica virgifera, including in insect populations resistant to B. thuringiensis. Some strains produce an exoprotease that kills the root-knot nematode Meloidogyne incognita and the soil nematode Caenorhabditis elegans. The A. faecalis N1-4 strain is capable of producing dimethyl disulfide and methyl isovalerate, which inhibit the growth of the fungus Aspergillus flavus and reduce the amount of mycotoxins in grain during storage (Ju et al., 2016; Gong et al., 2019; Pérez Ortega et al., 2021)

Achromobacter spp. The A. xylosoxidans soil strain causes 95 % mortality of larvae and 100 % mortality of adults of the housefly Musca domestica. Volatile esters (S-methylthiobutyrate, acetates) reduce the population of the gall nematode M. javanica and suppresse gall formation on tomatoes by 60 %. The endophytic strain CTA8689 reduces melon wilt caused by Fusarium oxysporum/F. solani by 60 % in a greenhouse thanks to siderophores and esterases (Yamaç et al., 2010; Dhaouadi et al., 2019; Deng et al., 2022; Mohamadpoor et al., 2022).

Brevundimonas spp. B. diminuta YYC02 produced 42 volatile compounds, of which butyl-2-methylbutanoate and isoamyl butyrate caused 90–100 % mortality of the root-knot nematode Meloidogyne javanica within 48 hours, while soil treatment reduced the number of galls by 37 % and increased the mass of cucumber shoots (Sun et al., 2023). Brevundimonas is part of the core microbiome of entomopathogenic nematodes (Steinernema, Meloidogyne) and actively adheres to the cuticle of J2 larvae, increasing their mortality and reducing egg hatching, which indirectly confirms the anti-PPN activity of the genus (Topalović et al., 2019). Although no classical Cry/Cyt toxins have been found in Brevundimona, a membrane organophosphate hydrolase has been studied in detail in B. diminuta, allowing the strain to use organophosphate insecticides as a source of phosphorus; the enzyme is localized in the periplasm by means of a Tat signal (Parthasarathy et al., 2016).

Leucobacter spp. Two strains, Verde1/Verde2, cause nematode death through a rare mechanism in which a sticky exopolymer sticks the nematode tails together into “stars”, leading to the death of the colony. Nematode resistance to these strains is controlled by individual genes, which emphasizes the specialized virulence of the genus. The L. aridicollis SASBG215 strain inhibits cucumber anthracnose Colletotrichum orbiculare and causes lysis of hyphae, presumably with the help of unknown polyketides (Hodgkin et al., 2013; Abdul Salam et al., 2022).

Ochrobactrum spp. Strain BS-206 synthesizes the glycolipid ochrozin, which kills 90–100 % of storage pests (Tribolium, Sitophilus, Callosobruchus) and has an insecticidal effect against the corn borer Spodoptera. The O. pseudogrignonense NC1 strain produces dimethyl disulfide and benzaldehyde, which cause up to 100 % mortality of young M. incognita individuals and reduce tomato gall formation by more than 60 % (Kumar et al., 2014; Yang T. et al., 2022).

As can be seen from the brief summary above, there is a variety of active substances for different pathogens, which can complicate mass analysis, but offer prospects for future research on little-studied endophytes that may have similar properties.

In this article, we presented 15 genomes of endophytic bacteria isolated from various parts of wheat, soybeans, and rapeseed. We have demonstrated the presence of genes responsible for insecticidal and fungicidal activity in the studied strains, with the largest number of genes encoding insecticidal and fungicidal proteins found in strains BZR 736, BZR 920, and BZR 936 of the genus Bacillus. However, for strains BZR 162, BZR 588, and BZR 201P of the Brucellaceae family, no genes encoding fungicidal proteins present in our database were identified

This may indicate several factors: firstly, bacteria of the genus Bacillus are more widely studied and published, and secondly, the database contains a bias in data on insecticidal and fungicidal proteins for this genus. Therefore, when interpreting the results obtained for less represented genera, we make the reservation that the presence of such proteins is possible in these genomes, but their sequences differ significantly from the publicly available sequences that we were able to aggregate into the database. Nevertheless, the bias in the data for the genus Bacillus does not affect the search for proteins with insecticidal and fungicidal properties. There is also little aggregated information in the literature on insecticidal and fungicidal proteins, which confirms the need to create databases of proteins associated with insecticidal and fungicidal properties

Nevertheless, the genomic data obtained in this study are consistent with the results of bioassays previously conducted for some of the strains studied. According to the initial screening of the bioresource collection, strains with pronounced entomopathogenic activity against the wax moth Galleria mellonella were identified. The strains BZR 1159, BZR 588, BZR 936, BZR 206, BZR 920, BZR 926, and BZR 277 (65–95 % mortality on the third day and 83–95 % on the fifth day) (Gyrnets (Bondarchuk), Asaturova, 2022).

With regard to the large mealworm beetle Tenebrio molitor, strains BZR 201, BZR 278, BZR 1159, BZR 635, BZR 762, BZR 736 showed themselves to be effective (72–98 % on the third day and 81–99 % on the fifth day). In addition, the potential multifunctionality of a number of strains was assessed: BZR 1159, BZR 936, and BZR G3, which showed insecticidal activity against the natural population of the codling moth Cydia pomonella, reaching 95.5 %, as well as fungicidal activity against apple disease pathogens of the genera Fusarium, Alternaria, Trichothecium, with mycelium inhibition of up to 84.8 % (Gyrnets (Bondarchuk), Asaturova, 2022; Gyrnets, Asaturova, 2023). The BZR 936 strain is worth noting: it has both insecticidal and fungicidal properties, which directly correlates with its identified lipopeptide synthase genes (iturin, fengycin, surfactin) and other biocontrol markers. Thus, comparison of the genomic composition with experimental biotests confirms that the presence of specific insecticidal protein genes and lipopeptide synthetase clusters is a reliable indicator of the biocontrol potential of strains. This opens up prospects for their further inclusion in programs for the development of biological products for the protection of agricultural crops.

## Conclusion

In this article, we present the taxonomic identification, assemblies, and annotations of 15 endophytic bacterial genomes, the samples of which were obtained from the roots and/or leaves of wheat, soybean, and rapeseed

Taxonomically, strains BZR 736, BZR 920, and BZR 936 belong to the genus Bacillus, strains BZR 635, BZR 466, BZR 206, and BZR 201 belong to the genus Leucobacter, and the remaining strains belong to the phylum Pseudomonadota. The phylogenetic tree constructed from a set of 120 bacterial marker genes fully reflects the topology of our taxonomic tree, which confirms the correctness of the established taxonomic identity at a relative level.

Genome assembly was performed in two stages: a preliminary stage of primary genome assembly and a hybrid (de novo + reference-guided) cascade approach based on a set of closely related references. Genome annotation was performed taking into account the databases of protein sequences with insecticidal properties prepared within the framework of this study, including the BPPRC database, and fungicidal properties, as well as the most complete known genome annotations of the corresponding species/genus

Analysis of the gene repertoire revealed differences between the analyzed strains and the reference genomes used. A high degree of correspondence between the gene repertoire of the studied strains and the corresponding reference genomes was shown by BZR 585 in relation to A. phenolicus, by BZR 762 and BZR 278 in relation to Alcaligenes sp., by BZR 588 and BZR 201P with respect to P. pullorum, by BZR 920 and BZR 936 in relation to B. velezensis, and by BZR 206, BZR 466 and BZR 201 with respect to L. aridicollis. This indicator may also confirm the correctness of the established taxonomic identity. At the same time, when compared with the closest reference genome, Leucobacter sp., BZR 635 shows only a small number of intersections, which may be due to both its distinctive taxonomic identity and the fragmentation of the genome assembly of this strain.

We demonstrated the presence of genes encoding fungicidal and insecticidal proteins in all strains except BZR 162, BZR 588, and BZR 201P. No genes encoding fungicidal proteins present in our database were identified for these strains. However, the results obtained in this study indicate that the strains under study, which possess a complex of lipopeptide synthetase and insecticidal toxin genes, demonstrate an experimentally confirmed broad spectrum of biological activity against insects and phytopathogens

Further targeted study of endophytic bacteria with fungicidal and insecticidal genes opens up prospects for identifying candidates for biocontrol agents of various pathogens and using bacteria to protect agricultural plants.

## Conflict of interest

The authors declare no conflict of interest.
